# Changing performance of surgical risk scores according to the endpoint of postoperative mortality in infective endocarditis patients

**DOI:** 10.3389/fcvm.2025.1543049

**Published:** 2025-03-13

**Authors:** Giuseppe Gatti, Antonio Fiore, Maria Ismail, Igor Vendramin, Alessandro Minati, Gianfranco Sinagra, Andrea Perrotti, Enzo Mazzaro

**Affiliations:** ^1^ Cardio-Thoracic and Vascular Department, University Hospital of Trieste, Trieste, Italy; ^2^ Department of Cardiac Surgery, Hôpitaux Universitaires Henri Mondor, Assistance Publique-Hôpitaux de Paris and Université Paris Est, Créteil, France; ^3^ Department of Thoracic and Cardio-Vascular Surgery, University Hospital Jean Minjoz and University of Franche-Comté, Besançon, France; ^4^ Department of Cardiac Surgery, Ospedale Santa Maria Della Misericordia, Udine, Italy; ^5^ Department of Clinical, Internal Medicine, Anesthesiology and Cardiovascular Sciences, La Sapienza University, Rome, Italy

**Keywords:** cardiac surgery, EuroSCORE, infective endocarditis, early postoperative mortality, endpoint, prediction, risk score

## Abstract

**Background:**

The optimal endpoint for reporting early mortality after cardiac operations for infective endocarditis (IE), as well as the optimal mortality target for surgical risk scores, are unresolved questions.

**Methods:**

Five risk scores created specifically to predict early mortality after cardiac operations for definite IE, and the European System for Cardiac Operative Risk Evaluation II, were assessed in terms of calibration, discrimination and accuracy in predicting early mortality following cardiac surgery for IE. The evaluation was based on five definite endpoints of postoperative mortality: In-hospital, 30-day, in-hospital/30-day, six-month, and one-year mortality. The six risk scores were tested in a population of 991 patients with definite IE who underwent 1,014 cardiac operations at five European university-affiliated centers.

**Results:**

There were 133 (13.1%) hospital deaths after surgery. Overall, 10% of patients died within 30 days after surgery, 10.4% of survivors died between 30 days and six months after surgery, and another 5.5% between six months and one year after surgery. All risk scores showed good prediction accuracy and at least acceptable discrimination for all endpoints of postoperative mortality. However, only one (IE-specific) risk score exhibited acceptable calibration for every endpoint of postoperative mortality.

**Conclusions:**

Since mortality decreases slowly throughout the first year after cardiac surgery for IE, it may be appropriate to report both in-hospital and one-year mortality (coupled endpoint) for this condition. For both endpoints, only one of the risk scores considered in this study showed acceptable calibration and discrimination.

## Introduction

Clinical trials exploring early postoperative outcomes in subjects undergoing cardiac operations for infective endocarditis (IE) either use in-hospital or 30-day mortality as an endpoint (1–3); combination of the two, namely in-hospital/30-day mortality, is sometimes adopted (4–6), and six-month mortality is occasionally reported (7). Accordingly, risk scores specifically devised to predict early mortality after surgery in the subset of patients with IE are developed and validated using one of the parameters above as mortality target. These risk scores are usually based on the patient's comorbidities, the complexity of planned surgery, and the nature of involved pathogen (2–7). This variability, however, impacts clinical decision-making and counseling the patients and their family, complicates the interpretation of outcomes and research comparability, and hinders the development of standardized guidelines.

To date there are no studies indicating which should be the optimal endpoint for reporting early mortality after cardiac surgery for IE. Consequently, from a methodological point of view, the optimal target of mortality for the IE-specific surgical risk scores remains an unresolved and urgent question. This is a significant issue since IE is a life-threatening disease that may require challenging surgery and cause higher rates of early postoperative complications than other less complex cardiac pathologies, as well as longer hospital stays and higher costs of health care. In addition, the timing of onset of complications after surgery for IE can also be very different. It is known indeed that severe complications such as surgically related infections may appear late after surgery and that, unfortunately, recurrence of infection in patients operated on for IE is not such a rare event. By convention, every surgical site infection that occurs within one year from surgery should be considered related to that surgery, at least until proven otherwise (1). Therefore, the present authors wondered whether the same endpoints of mortality that are usually adopted for other heart diseases of surgical interest might also be used for IE.

The aim of the present short communication is to show any changes in the performance of six risk scores in predicting early mortality following cardiac operations for IE based on five definite endpoints of postoperative mortality: In-hospital, 30-day, in-hospital/30-day, six-month, and one-year mortality. The risk scores were evaluated about calibration, discrimination, and accuracy of prediction.

## Methods

In a clinical investigation by the present authors, which has been published recently in *The American Heart Journal* (8), five risk scores created specifically and selectively to predict early mortality after cardiac surgery for definite IE—STS-IE (Society of Thoracic Surgeons for IE) (2); PALSUSE (Prosthetic valve, Age ≥70, Large intra-cardiac destruction, *Staphylococcus spp*, Urgent surgery, Sex [female], logistic EuroSCORE ≥10 (3); ANCLA (Anemia, New York Heart Association functional class IV, Critical state, Large intra-cardiac destruction, surgery on thoracic Aorta) (4); AEPEI II (Association pour l’Étude et la Prévention de l’Endocardite Infectieuse II) (5); and APORTEI (A*n*álisis de los factores PROnósticos en el Tratamiento quirúrgico de la Endocarditis Infecciosa) (6) (Supplementary Tables S1–S3) –, as well as the European System for Cardiac Operative Risk Evaluation II (EuroSCORE II) (9), which has been widely and commonly used throughout Europe since 2012 to evaluate the mortality risk after any type of cardiac operation, were validated and compared in terms of calibration, discrimination and accuracy in predicting 30-day postoperative mortality. The above risk scores were selected regarding their acceptance in Literature, clinical applicability, and availability of the collected data (8).

In the present study, the same risk scores are now evaluated according to the endpoint of early postoperative mortality. The study patients, definitions and statistical methods are approximately the same as in the previously cited study from the authors (8). Baseline characteristics, surgical and IE-related features, and early postoperative deaths of 991 adults (20 years of age or older) with definite IE who underwent 1,014 cardiac operations at five European university-affiliated centers were prospectively recorded in computerized databases. Only the cases with known pathogen, without missing values for all considered variables, and having at least one-year of follow-up were retained for the present retrospective analyses. The patients underwent surgery for IE during different time periods depending on the center. The series of cases were consecutive for all but one center. The five series were merged and managed as one single cohort. Definite IE was defined according to the 2023 Duke-International Society for Cardiovascular Infectious Diseases criteria (1). For each risk score's computation, the definitions of variables used were those reported in the corresponding original paper (2–6, 9). A mix of definitions of variables deriving from the six risk scores considered was adopted for reporting data in the present manuscript. The in-hospital death was defined as death occurring before hospital discharge; the 30-day death was defined as death occurring within the postoperative day 30 (regardless of hospital discharge); the in-hospital/30-day death was defined as death occurring before hospital discharge, or within the postoperative day 30 for discharged patients; the six-month and one-year deaths were defined as deaths occurring within six months and one year after surgery, respectively. The causes of death were not investigated.

An informed consent for future retrospective studies was obtained preoperatively from each patient. The study protocol conforms to the ethical guidelines of the 1975 Declaration of Helsinki as reflected in *a priori* approval by the human research committee of each participating institution.

### Statistical methods

Data is presented as absolute number, percentage, or mean ± standard deviation. Calibration of each risk score was assessed using calibration plot analysis. Initially, a regression line (linear trendline) was derived from plot data of each risk score. The regression line was estimated using the observed event rates as a dependent variable and, as an independent variable, the estimated probabilities by the score. Slope and intercept were derived from equation of each regression line and used to compare the risk scores to each other. For intercept-values closer to 0, the closer to 1 the slope-value, the higher the calibration of the risk score. For identical or very similar slope-values, the closer to 0 the intercept-value, the higher the risk score calibration. Calibration was defined as acceptable when the following conditions were satisfied simultaneously: (1) Slope-values range between 0.7 and 1.3; (2) Intercept-values range between −0.1 and 0.1. Later, a regression curve (polynomial trendline) was obtained to evaluate semi-quantitatively each risk score. The curve was estimated using the observed event rates as a dependent variable and, as an independent variable, the estimated probabilities by the score. The order of equation and value of intercept were arbitrarily set to 6 and 0, respectively. The square of the correlation coefficient r (*R^2^* or R-squared) was adopted to measure how well the trendline approximated the plot data (*R^2^*-values range from 0 to 1, with higher values indicating a better fit). Both linear and polynomial trendline approximated well the plot data for *R^2^*-values >0.7. Afterwards, a receiver-operating characteristic curve was plotted for each risk score and the area under the curve (AUC) was calculated as a measure of discrimination of the model (the possible values of AUC range from 0.5, no discrimination, to 1, perfect discrimination). Finally, accuracy of prediction of each risk score was evaluated with the ratio between the observed and the expected mortality (observed-to-expected ratio, O/E), and the Brier's score (scores are bound between 0 and 1, with 0 indicating perfect accuracy and 1 perfect inaccuracy). Acquisition of the data entries was performed using Microsoft Office Excel, version 2007. Data analysis was performed using the SPSS software package for Windows, version 13.0.

## Results

### Early postoperative mortality

There were 133 (13.1%) hospital deaths after surgery (only 100 within postoperative day 30). One patient died after hospital discharge but within the postoperative day 30. Out of discharged patients, 7.1% died within six months from surgery, and 12.3% within one year. Overall, 10% of patients died within 30 days after surgery, 10.4% of survivors died between 30 days and six months after surgery, and another 5.5% between six months and one year after surgery. There were 23 (2.3%) cases of reoperation due to recurrence of cardiac infection; two patients died within 30 days from surgery, one patient within six months, and another one within one year (Table 1).

**Table 1:** Perioperative variables frequency in the overall series of cases (*N* = 1,014)^a,b^.

Variable	*N*	*%*
No. of patients	991 ^c^	97.7
Age, years		
22–49	168	16.6
50–59	181	17.9
60–69	296	29.2
70–79	283	27.9
80–89	85	8.4
90 or more	1	0.1
Female gender	240	23.7
Arterial hypertension	529	52.2
Diabetes mellitus	228	22.5
Insulin-dependent	47	4.6
Non-insulin-dependent	181	17.9
Anemia	908	89.6
Renal failure	298	29.4
Chronic lung disease	81	8.0
Previous cardiac surgery	334	32.9
CABG	23	2.3
Valve surgery	311	30.7
NYHA functional class		
I–II	593	58.5
III–IV	421 ^d^	41.5
Cardiogenic shock	98	9.7
Life-threatening arrhythmias	132	13.0
Critical state	230	22.7
Urgent surgical priority	528	52.1
Active IE	1,003	98.9
Urgent or emergency status, no cardiogenic shock	440	43.4
Emergency, salvage, or cardiogenic shock	88	8.7
Type of involved heart valve		
Native	703	69.3
Prosthetic	311	30.7
No. of involved heart valves		
One	760	75.0
Two or more	254	25.0
Paravalvular abscess	355	35.0
Large intra-cardiac destruction	508	50.1
Concomitant surgery of thoracic aorta	67	6.6
Involved pathogen		
*Staphylococcus aureus*	242	23.9
Other than *Staphylococcus aureus*	772	76.1
Early postoperative death		
In-hospital	133	13.1
30-Day	101	10.0
In-hospital/30-Day	134	13.2
Six-month	196	19.3
One-year	241	23.8

CABG, coronary artery bypass grafting; IE, infective endocarditis; NYHA, New York Heart Association.

^a^ Study patients.

^b^ See text and *Ref.* (8) for the adopted definitions of perioperative variables.

^c^ Twenty-two patients underwent two consecutive cardiac operations, both for IE; one patient had three consecutive cardiac operations, all owing to IE.

^d^ NYHA functional class IV was present in 196 (19.3%) cases.

### Risk score performance

**Figure 1:**
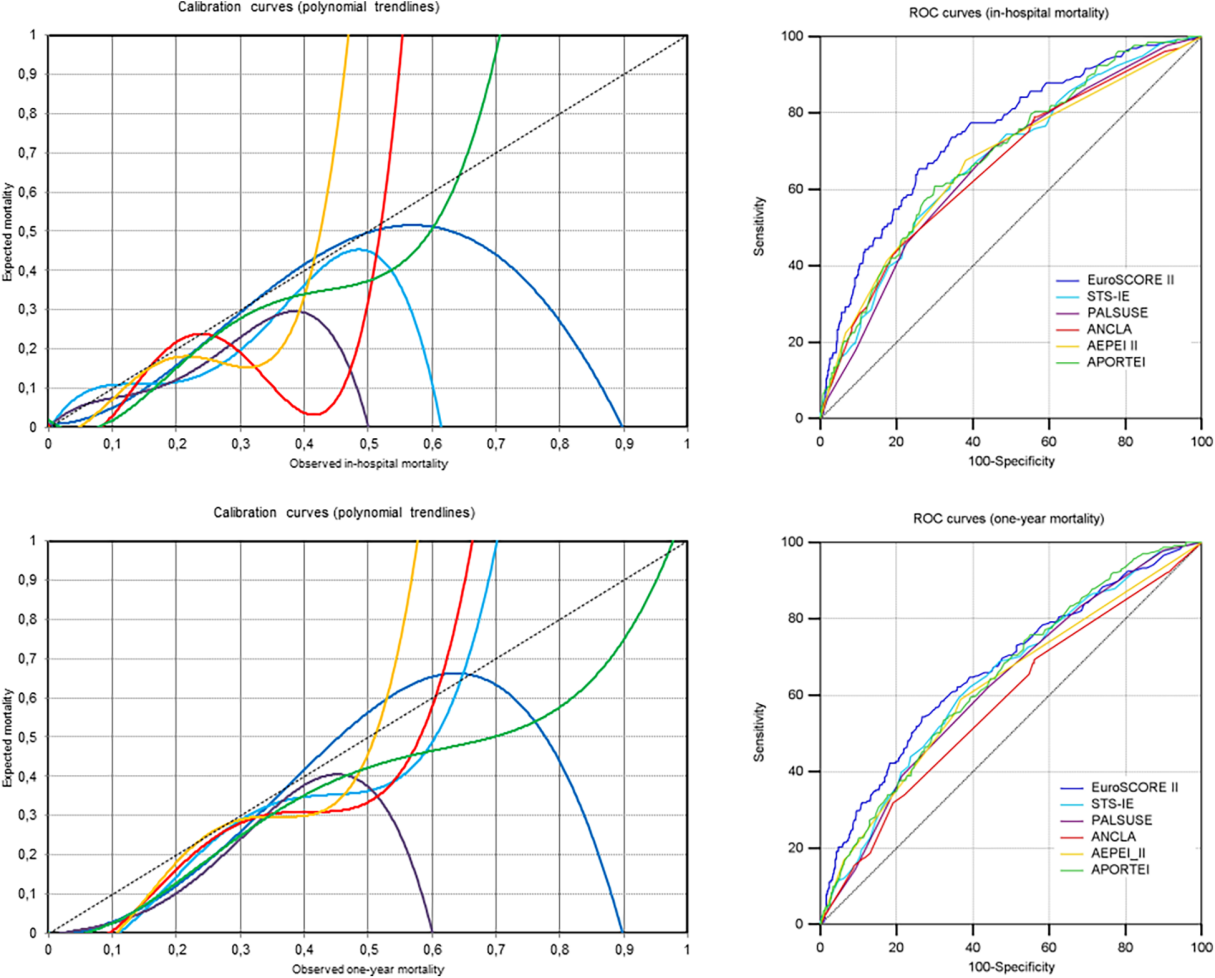
Calibration and discrimination of the six risk scores in study patients for in-hospital and one-year mortality (i.e., the two components of the proposed combined endpoint for reporting early mortality after cardiac surgery for IE) are shown here using calibration and ROC curves, respectively.

Calibration of the six risk scores for the five definite endpoints of postoperative mortality is summarized in Table 2. While the AEPEI II and APORTEI scores showed acceptable calibration for in-hospital mortality, the STS-IE, ANCLA and AEPEI II scores showed acceptable calibration for one-year mortality. Only the AEPEI score II showed acceptable calibration for all endpoints (Table 2, Figure 1, and Supplementary Figures S1, S2).

**Table 2:** Calibration of the six risk scores for the five definite endpoints of postoperative mortality (*N* = 1,014)^a,b^.

Risk score	Regression line method					Regression curve method				
	Endpoint of mortality					Endpoint of mortality				
	In-hospital	30-Day	In-hospital/30-day	Six-month	One-year	In-hospital	30-day	In-hospital/30-day	Six-month	One-year
EuroSCORE II	NA	NA	NA	NA	NA	Poor	Poor	Poor	Poor	Poor
STS-IE	NA	Acceptable	Poor	Acceptable	Acceptable	Poor	Poor	Acceptable	Acceptable	Acceptable
PALSUSE	NA	NA	NA	NA	NA	Poor	Poor	Poor	Poor	Poor
ANCLA	NA	Acceptable	NA	Poor	Acceptable	Poor	Poor	Poor	Poor	Acceptable
AEPEI II	Acceptable	Acceptable	Acceptable	Acceptable	Acceptable	Acceptable	Acceptable	Acceptable	Acceptable	Acceptable
APORTEI	Acceptable	Poor	Acceptable	Acceptable	Acceptable	Acceptable	Acceptable	Acceptable	Poor	Poor

AEPEI, Association pour l'Étude et la Prévention de l'Endocardite Infectieuse; ANCLA, Anemia, New York Heart Association functional class IV, critical state, large intra-cardiac destruction, surgery on thoracic Aorta; APORTEI, Análisis de los factores PROnósticos en el Tratamiento quirúrgico de la Endocarditis Infecciosa; EuroSCORE, European System for Cardiac Operative Risk Evaluation; NA, not appropriate (trendline does not approximate well the plot data); PALSUSE, Prosthetic valve, Age =70, Large intracardiac destruction, Staphylococcus spp, Urgent surgery, Sex (female), EuroSCORE =10; STS-IE, Society of Thoracic Surgeons-Infective Endocarditis.

^a^ Study patients.

^b^ See text for the adopted definitions.

Four risk scores (EuroSCORE II, PALSUSE, ANCLA and APORTEI) showed the highest AUC values for 30-day mortality, and two risk scores (STS-IE and AEPEI II) for in-hospital/30-day mortality. For all risk scores, the lowest AUC values were observed for one-year mortality, though the lower limit of the 95% confidence interval of AUC was always above 0.5 (0.548 or greater) (Figure 1 and Supplementary Figures S1, S2).

Overall, O/E was always equal to, or greater than, 0.928 and equal to, or less than, 1.023, for all risk scores and for all mortality endpoints; Brier's score was 0.179 or less. For all but one risk score, the best predictive accuracy was observed for one-year mortality, at least based on O/E (O/E closer to 1); the AEPEI score II showed the best O/E for 30-day mortality; the PALSUSE score showed the highest predictive accuracy both for six-month and one-year mortality. According to Brier's score instead, the best predictive accuracy was observed for 30-day mortality for all risk scores (Supplementary Figure S1).

## Comment

Six surgical risk scores were evaluated in this study. Their main characteristics are reported in Supplementary Tables S1–S3 and summarized below. EuroSCORE II consists of 18 variables and has been modeled from a contemporary surgical cohort of 22,381 patients, including 497 (2.2%) with active IE (9). It has been created to predict 30-day mortality after any cardiac operation. Since its publication (2012) it was quickly and widely introduced into clinical practice in Europe. The STS-IE score has been published in 2011 (2). It derives from a series of 13,619 IE-patients who had been operated on at 824 centers in North America between 2002 and 2008. It includes 13 variables: nine host-related factors, two heart-related factors, and two extracardiac events. It is routinely used in North America. The PALSUSE score has been published in 2014 (3). It derives from a series of 437 IE-patients who had been operated on at 26 Spanish hospitals between 2008 and 2010. It includes seven variables: three host-related, two heart-related, one pathogen-related, and logistic EuroSCORE ≥10%. The ANCLA score has been published in 2017 (4). It derives from a series of 138 IE-patients who had operated on at one-single Italian university hospital between 1999 and 2015. It includes two host-related variables, one heart-related variable, one laboratory finding, and one surgical data. The AEPEI score II also has been published in 2017 (5). It is the three-variable alternate model of AEPEI score I. Both risk scores derive from a series of 361 consecutive patients who had undergone surgery for IE in one Italian (period, 1999–2015) and seven French (throughout 2008) cardiac surgery centers. The AEPEI score II includes only two host-related variables and one extra-cardiac event. Unlike the three previous risk scores, which derive directly from a multivariable analysis, the APORTEI score derives from a meta-analysis performed on 16 selected studies (publication years, 2007–2018) comprising 7,484 IE-patients (6). The APORTEI score includes 11 variables: six host-related factors, three heart-related factors, one extra-cardiac event, and one pathogen-related factor.

According to the statistical analyses of the present study, (1) all risk scores showed good accuracy of prediction and acceptable discrimination for every index endpoint, though the highest discrimination was for death occurring before hospital discharge and/or within 30 days from surgery; (2) EuroSCORE II had a good discrimination for early postoperative mortality, though its calibration was poor (8); (3) The AEPEI score II showed an acceptable calibration for all endpoints of early postoperative mortality; (4) While the AEPEI II and APORTEI scores showed acceptable calibration for in-hospital mortality, the STS-IE, ANCLA and AEPEI II scores showed acceptable calibration for one-year mortality.

Hence, based on these results and those of previous studies (8, 10), EuroSCORE II should be the recommended risk model to discriminate mortality that occurs in IE-patients immediately after cardiac surgery. However, since its calibration is poor and generally lower than IE-specific risk scores, EuroSCORE II should be accompanied by specific risk scores such as the AEPEI II or the APORTEI score. In addition, the STS-IE, ANCLA, and AEPEI II scores, which showed acceptable discrimination and calibration one year after surgery, should be adopted to identify patients who need to be carefully monitored after hospital discharge due to the high risk of IE recurrence.

The authors of this study intend to propose a coupled endpoint—in-hospital & at one year from surgery—to report mortality after cardiac operations for IE. This original approach aims to not underestimate the true impact of cardiac surgery in IE-patients and derives from the following interdependent concepts: (1) Either in IE-patients treated with medical therapy alone or in combination with surgery, the mortality rate declines slowly throughout the first year after hospital discharge; (2) In IE-patients, it is reasonable to distinguish between immediate and early postoperative death, since the causes and mechanisms underlying the two types of death are different. In fact, complications after surgery for IE are time-dependent in nature. While immediate postoperative deaths are usually related to the host's preoperative comorbidity and nutritional status, heart failure due to extensive cardiac destruction that requires complex surgical reconstruction, pathogen virulence, and uncontrolled infection, early postoperative deaths are generally related to patient's failure to recover from surgical stress and recurrence of infection caused by incomplete surgical cleanup or failure of antibiotic treatment (7, 11, 12). These two peculiar aspects of IE-surgery explain at least partially why it can be very different from surgery for other heart diseases, pose more complex challenges and require original and diversified approaches.

In conclusion, since mortality decreased slowly throughout the first year after surgery, also for patients in this study, from a methodological point of view it may be appropriate for IE-patients undergoing cardiac surgery to use a coupled endpoint and report both in-hospital and one-year mortality. This original approach could provide a more accurate picture of the outcomes of surgery for IE.

## Data Availability

The raw data supporting the conclusions of this article will be made available by the authors, without undue reservation.

## Supplementary material

Supplementary Figure S1 Changes in calibration, discrimination and accuracy in predicting in-hospital (In-H), 30-day (30-D), in-hospital/30-day (In-H/30-D), six-month (6-M) and one-year (1-Y) mortality after surgery for IE of six risk scores (*N* = 1,014).

Supplementary Figure S2 Calibration curves for in-hospital, 30-day, in-hospital/30-day, six-month and one-year mortality after surgery for IE of six risk scores (*N* = 1,014).

Supplementary Figure S3 Receiver-operating characteristics (ROC) curves for in-hospital, 30-day, in-hospital/30-day, six-month and one-year mortality after surgery for IE of six risk scores (*N* = 1,014).
