# Supplementary material for: Changing performance of surgical risk scores according to the endpoint of postoperative mortality in infective endocarditis patients
Source: Front Cardiovasc Med. 2025 Mar 13;12:1543049. doi: 10.3389/fcvm.2025.1543049 (PMC11965892; doi:10.3389/fcvm.2025.1543049)
Supplement: Supplementary Figure S1 — Changes in calibration, discrimination and accuracy in predicting in-hospital (In-H), 30-day (30-D), in-hospital/30-day (In-H/30-D), six-month (6-M) and one-year (1-Y) mortality after surgery for IE of six risk scores (N = 1,014). [file Image1.pdf]

Supplementary Figure S1

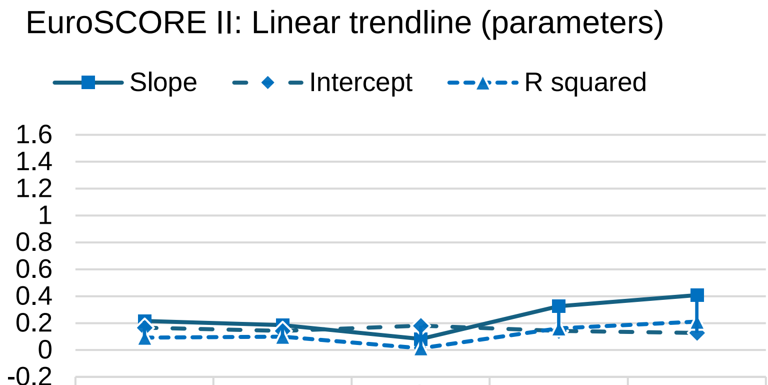

	In-H	30-D	In-H/30-D	6-M	1-Y
Slope	0.216	0.186	0.082	0.325	0.408
Intercept	0.167	0.143	0.181	0.142	0.127
R squared	0.091	0.1	0.013	0.162	0.211

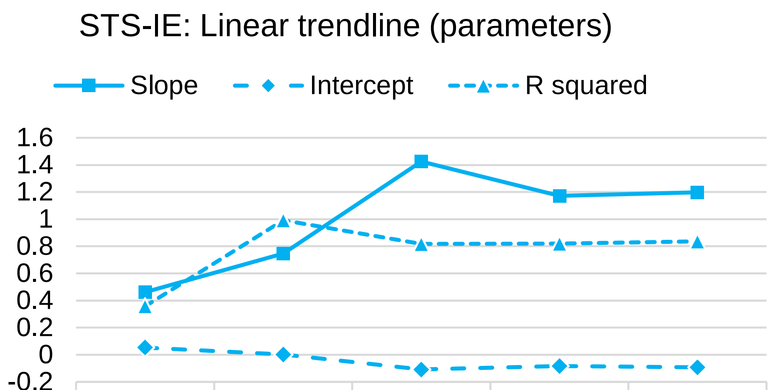

	In-H	30-D	In-H/30-D	6-M	1-Y
Slope	0.462	0.747	1.425	1.173	1.199
Intercept	0.053	0.003	-0.108	-0.084	-0.093
R squared	0.358	0.992	0.817	0.821	0.836

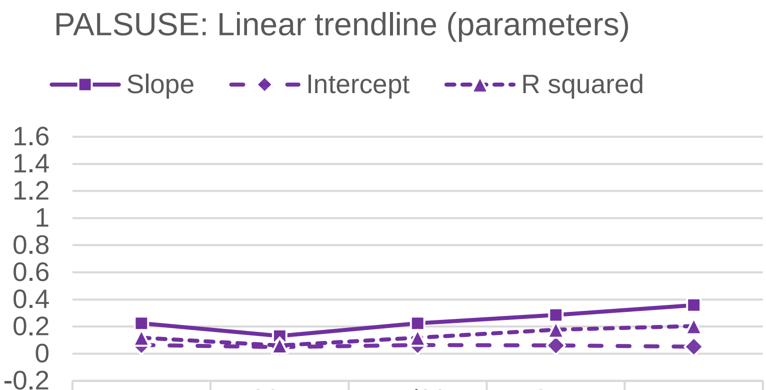

	In-H	30-D	In-H/30-D	6-M	1-Y
Slope	0.224	0.131	0.224	0.285	0.358
Intercept	0.064	0.049	0.064	0.061	0.053
R squared	0.118	0.061	0.118	0.178	0.204

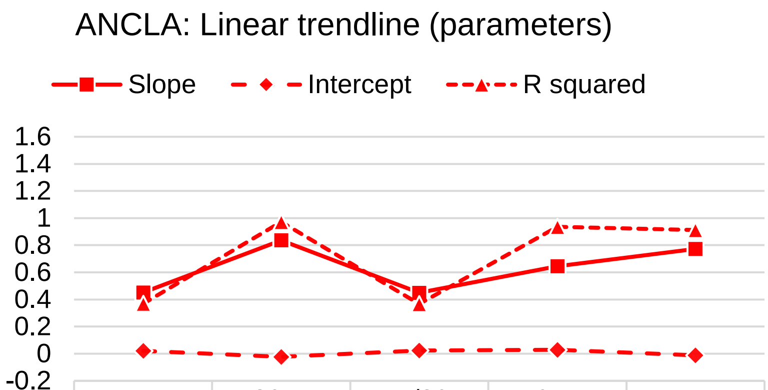

	In-H	30-D	In-H/30-D	6-M	1-Y
Slope	0.451	0.836	0.45	0.646	0.772
Intercept	0.022	-0.023	0.023	0.029	-0.011
R squared	0.369	0.972	0.367	0.937	0.913

AEPEI II: Linear trendline (parameters)

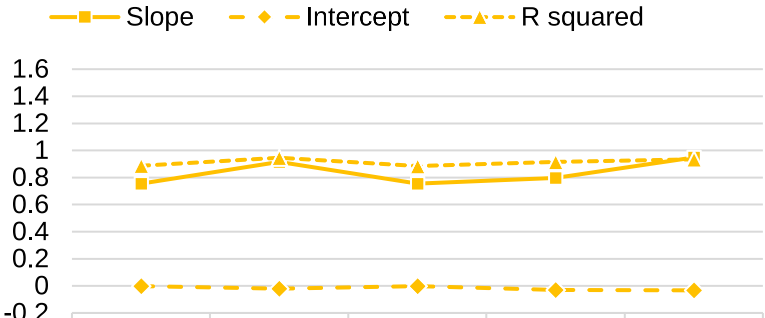

	In-H	30-D	In-H/30-D	6-M	1-Y
Slope	0.755	0.916	0.755	0.798	0.948
Intercept	-0.002	-0.02	-0.002	-0.03	-0.033
R squared	0.887	0.946	0.885	0.915	0.934

APORTEI: Linear trendline (parameters)

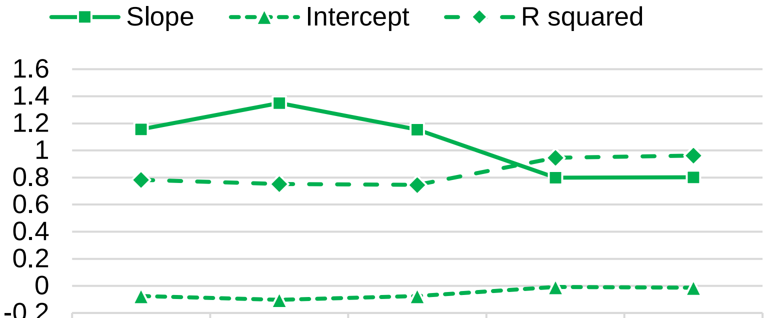

	In-H	30-D	In-H/30-D	6-M	1-Y
Slope	1.156	1.35	1.154	0.799	0.802
Intercept	-0.074	-0.103	-0.076	-0.008	-0.013
R squared	0.783	0.752	0.746	0.946	0.962

EuroSCORE II: Area under the ROC curve

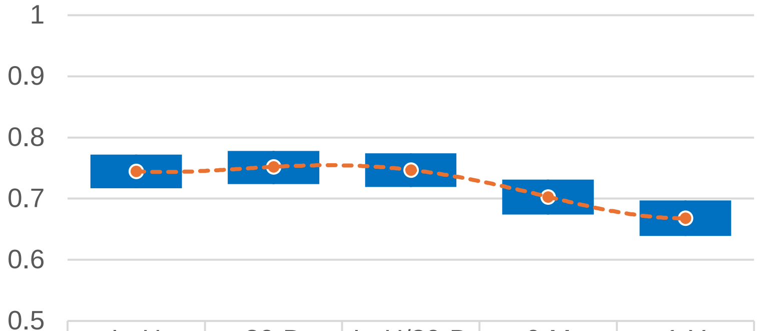

	In-H	30-D	In-H/30-D	6-M	1-Y
Upper limit	0.772	0.778	0.774	0.731	0.697
AUC	0.745	0.752	0.747	0.703	0.668
Lower limit	0.717	0.724	0.719	0.674	0.639

STS-IE: Area under the ROC curve

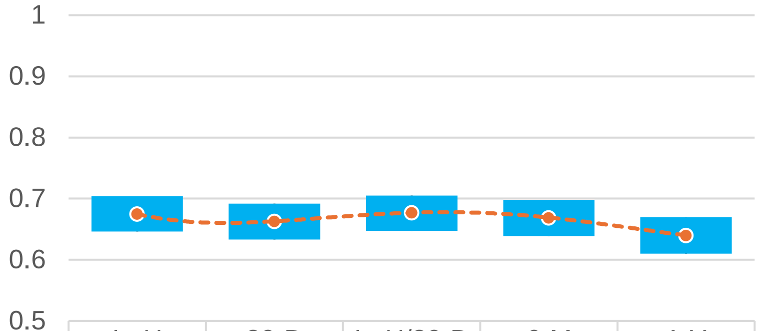

	In-H	30-D	In-H/30-D	6-M	1-Y
Upper limit	0.704	0.692	0.705	0.698	0.67
AUC	0.675	0.663	0.677	0.669	0.64
Lower limit	0.646	0.633	0.647	0.639	0.61

PALSUSE: Area under the ROC curve

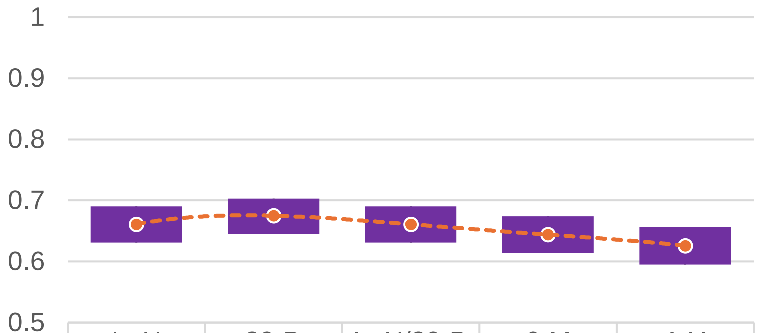

	In-H	30-D	In-H/30-D	6-M	1-Y
Upper limit	0.69	0.703	0.69	0.674	0.656
AUC	0.661	0.675	0.661	0.644	0.626
Lower limit	0.631	0.645	0.631	0.614	0.595

ANCLA: Area under the ROC curve

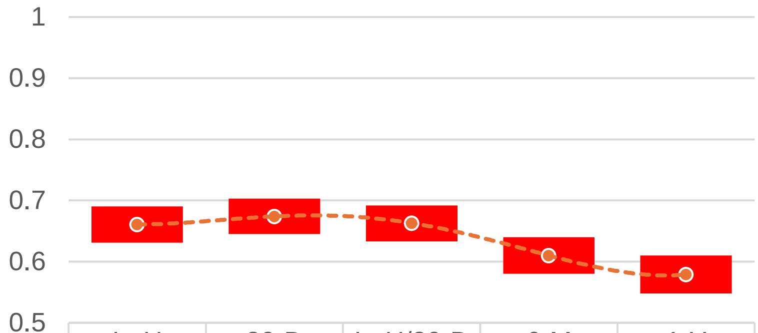

	In-H	30-D	In-H/30-D	6-M	1-Y
Upper limit	0.69	0.703	0.692	0.64	0.61
AUC	0.661	0.674	0.663	0.61	0.579
Lower limit	0.631	0.645	0.633	0.58	0.548

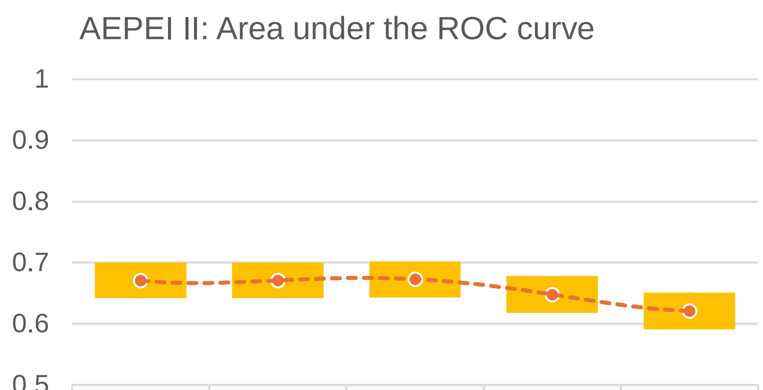

	In-H	30-D	In-H/30-D	6-M	1-Y
Upper limit	0.7	0.7	0.702	0.678	0.651
AUC	0.671	0.671	0.673	0.648	0.621
Lower limit	0.642	0.642	0.643	0.618	0.591

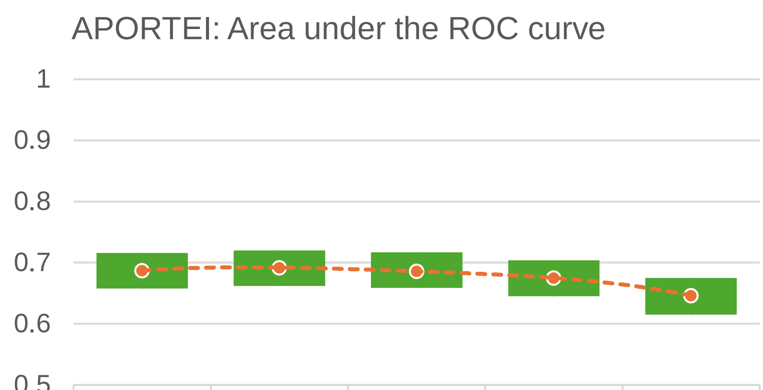

	In-H	30-D	In-H/30-D	6-M	1-Y
Upper limit	0.716	0.72	0.717	0.704	0.675
AUC	0.687	0.692	0.686	0.675	0.646
Lower limit	0.658	0.662	0.659	0.645	0.615

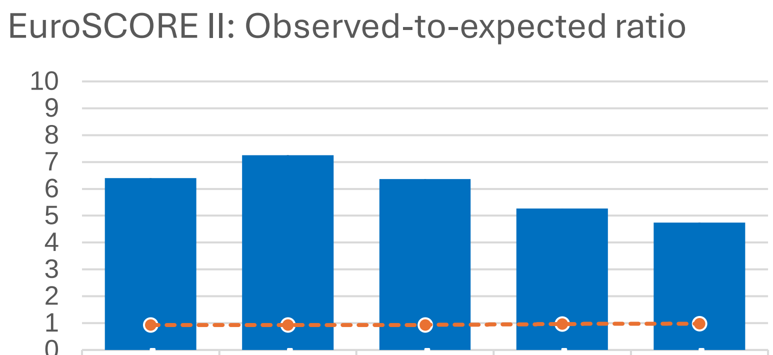

	In-H	30-D	In-H/30-D	6-M	1-Y
Upper limit	6.402	7.252	6.367	5.264	4.739
O/E	0.934	0.928	0.934	0.965	0.98
Lower limit	0	0	0	0	0

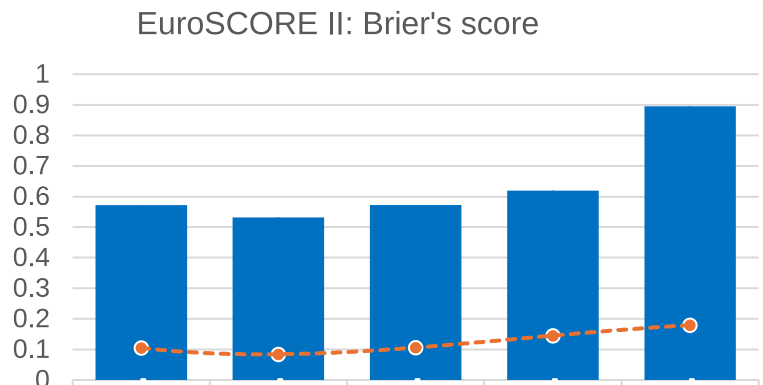

	In-H	30-D	In-H/30-D	6-M	1-Y
Upper limit	0.571	0.531	0.572	0.62	0.895
Score	0.105	0.084	0.106	0.145	0.179
Lower limit	0	0	0	0	0

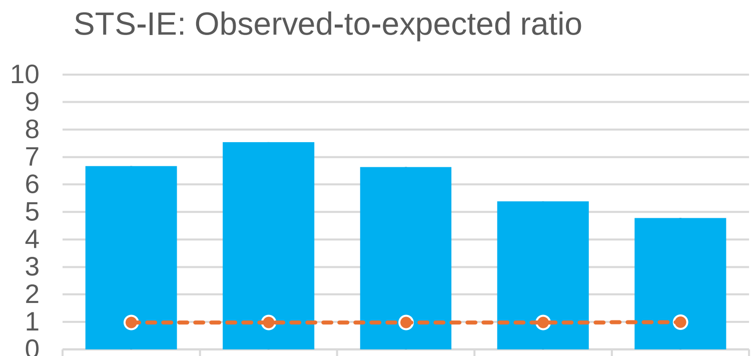

	In-H	30-D	In-H/30-D	6-M	1-Y
Upper limit	6.67	7.542	6.64	5.382	4.78
O/E	0.981	0.982	0.98	0.98	0.99
Lower limit	0	0	0	0	0

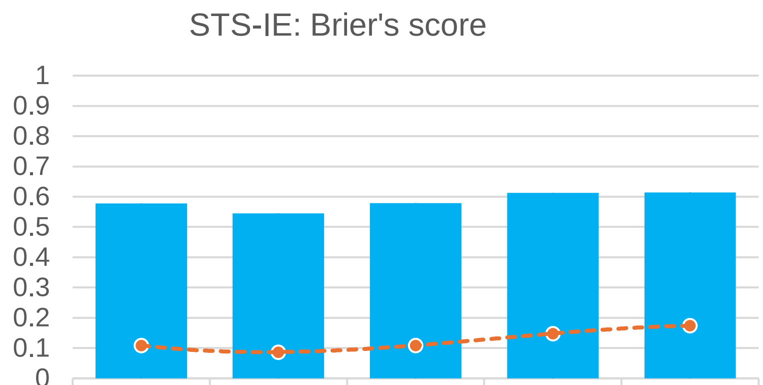

	In-H	30-D	In-H/30-D	6-M	1-Y
Upper limit	0.578	0.545	0.579	0.613	0.614
Score	0.109	0.087	0.109	0.148	0.174
Lower limit	0	0	0	0	0

PALSUSE: Observed-to-expected ratio

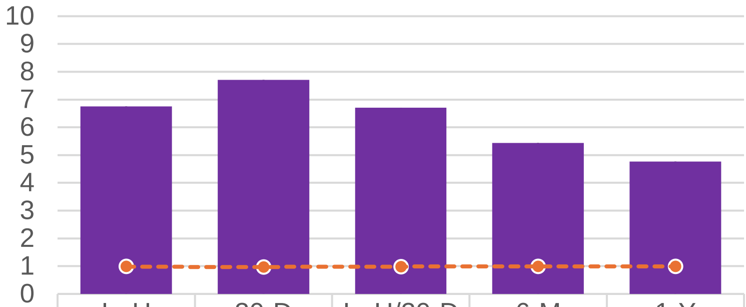

	In-H	30-D	In-H/30-D	6-M	1-Y
Upper limit	6.747	7.707	6.705	5.432	4.771
O/E	0.987	0.973	0.985	0.991	0.991
Lower limit	0	0	0	0	0

PALSUSE: Brier's score

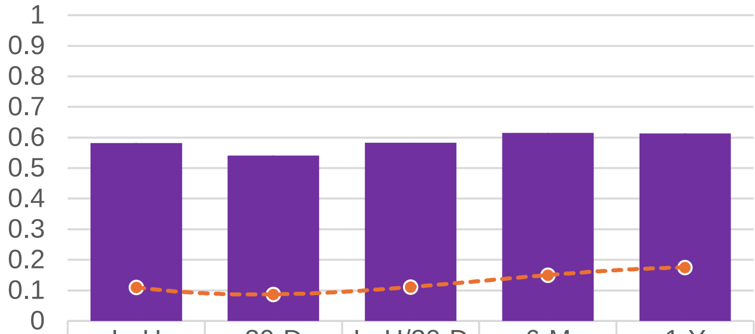

	In-H	30-D	In-H/30-D	6-M	1-Y
Upper limit	0.582	0.541	0.583	0.615	0.613
Score	0.11	0.087	0.111	0.15	0.175
Lower limit	0	0	0	0	0

ANCLA: Observed-to-expected 99999ratio

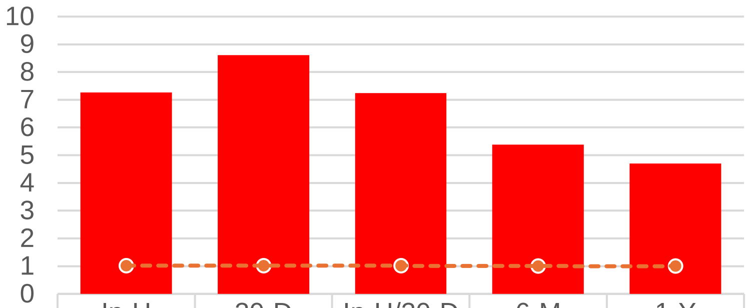

	In-H	30-D	In-H/30-D	6-M	1-Y
Upper limit	7.266	8.61	7.241	5.379	4.702
O/E	1.015	1.023	1.014	1.003	1.002
Lower limit	0	0	0	0	0

ANCLA: Brier's score

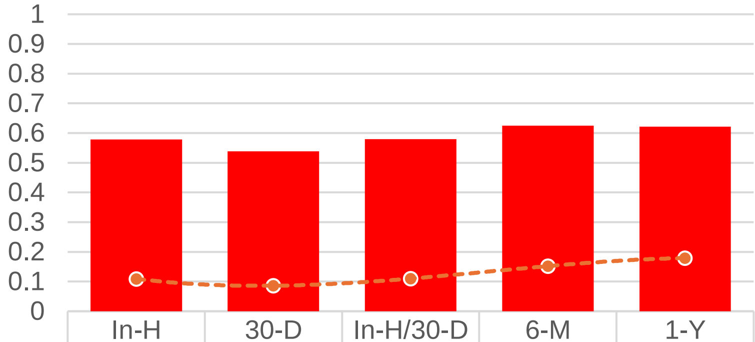

	In-H	30-D	In-H/30-D	6-M	1-Y
Upper limit	0.579	0.539	0.58	0.625	0.622
Score	0.109	0.086	0.11	0.152	0.179
Lower limit	0	0	0	0	0

AEPEI II: Observed-to-expected ratio

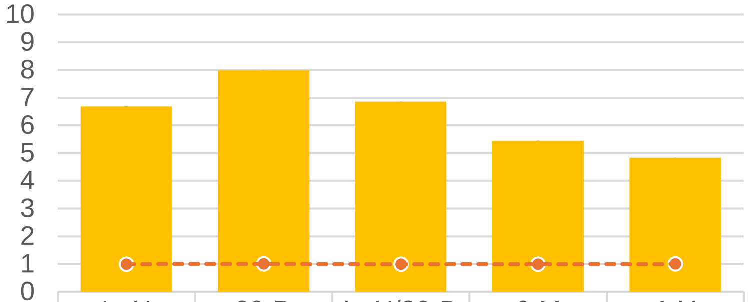

	In-H	30-D	In-H/30-D	6-M	1-Y
Upper limit	6.684	7.983	6.856	5.448	4.832
O/E	0.994	0.999	0.993	0.991	0.998
Lower limit	0	0	0	0	0

AEPEI II: Brier's score

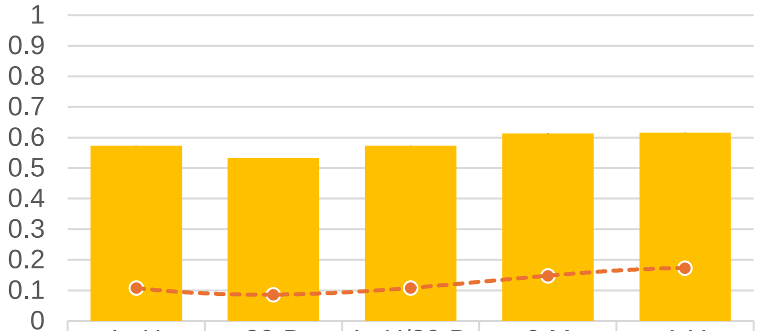

	In-H	30-D	In-H/30-D	6-M	1-Y
Upper limit	0.573	0.534	0.573	0.613	0.616
Score	0.108	0.086	0.108	0.148	0.173
Lower limit	0	0	0	0	0

APORTEI: Observed-to-expected ratio

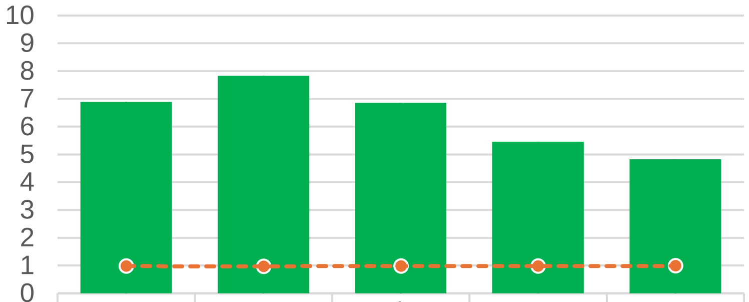

	In-H	30-D	In-H/30-D	6-M	1-Y
Upper limit	6.892	7.83	6.86	5.461	4.82
O/E	0.982	0.972	0.98	0.978	0.988
Lower limit	0	0	0	0	0

APORTEI: Brier's score

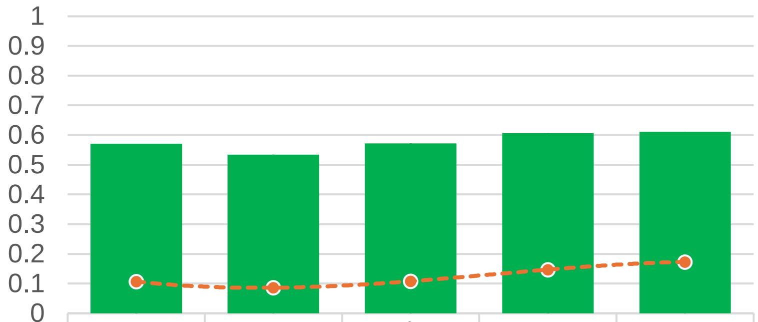

	In-H	30-D	In-H/30-D	6-M	1-Y
Upper limit	0.571	0.534	0.572	0.607	0.611
Score	0.107	0.086	0.108	0.147	0.173
Lower limit	0	0	0	0	0
