# Supplementary material for: Changing performance of surgical risk scores according to the endpoint of postoperative mortality in infective endocarditis patients
Source: Front Cardiovasc Med. 2025 Mar 13;12:1543049. doi: 10.3389/fcvm.2025.1543049 (PMC11965892; doi:10.3389/fcvm.2025.1543049)

Suppl. Figure S2

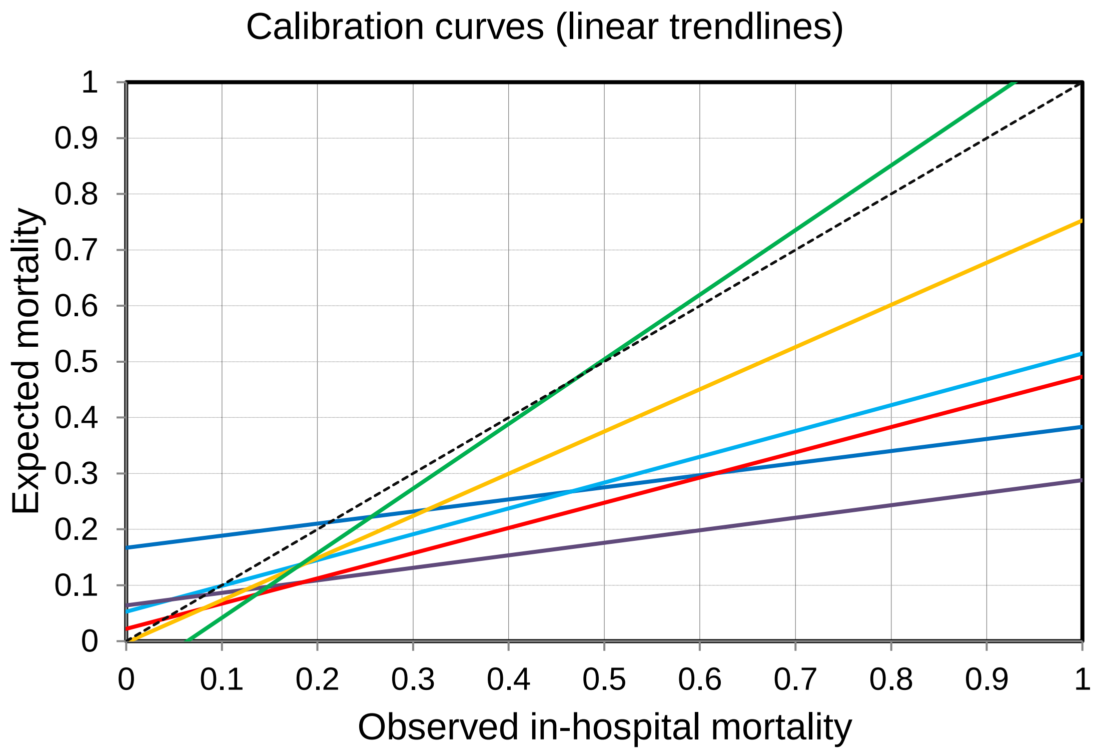

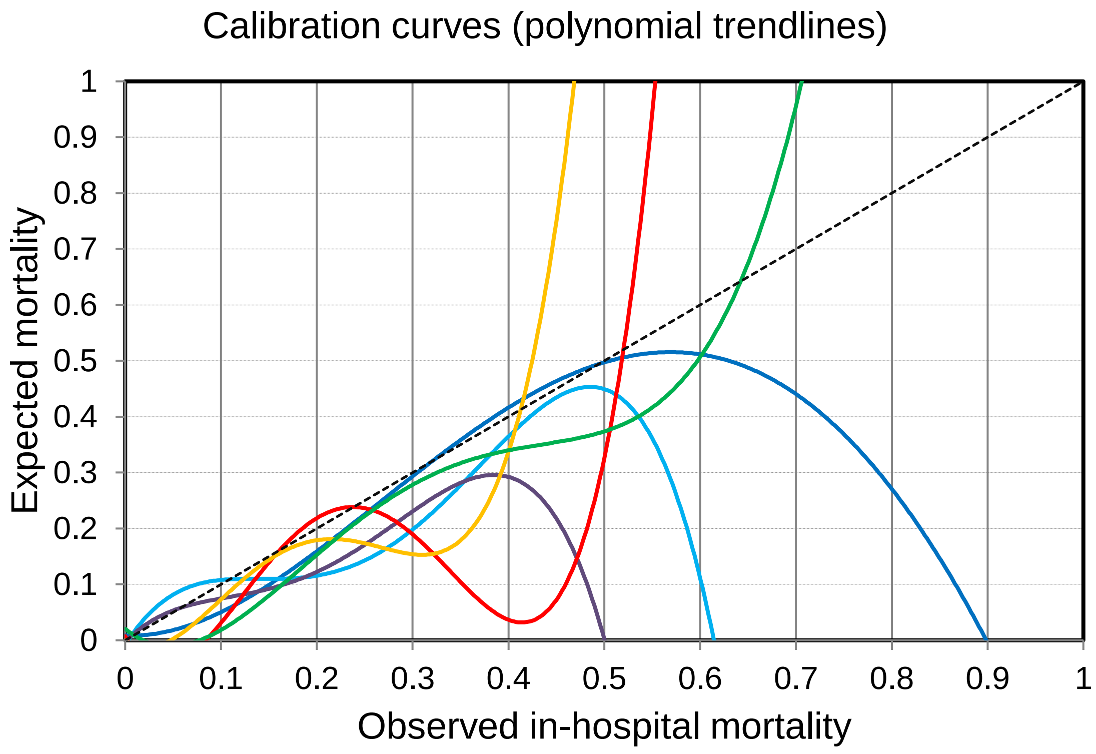

Suppl. Figure S2

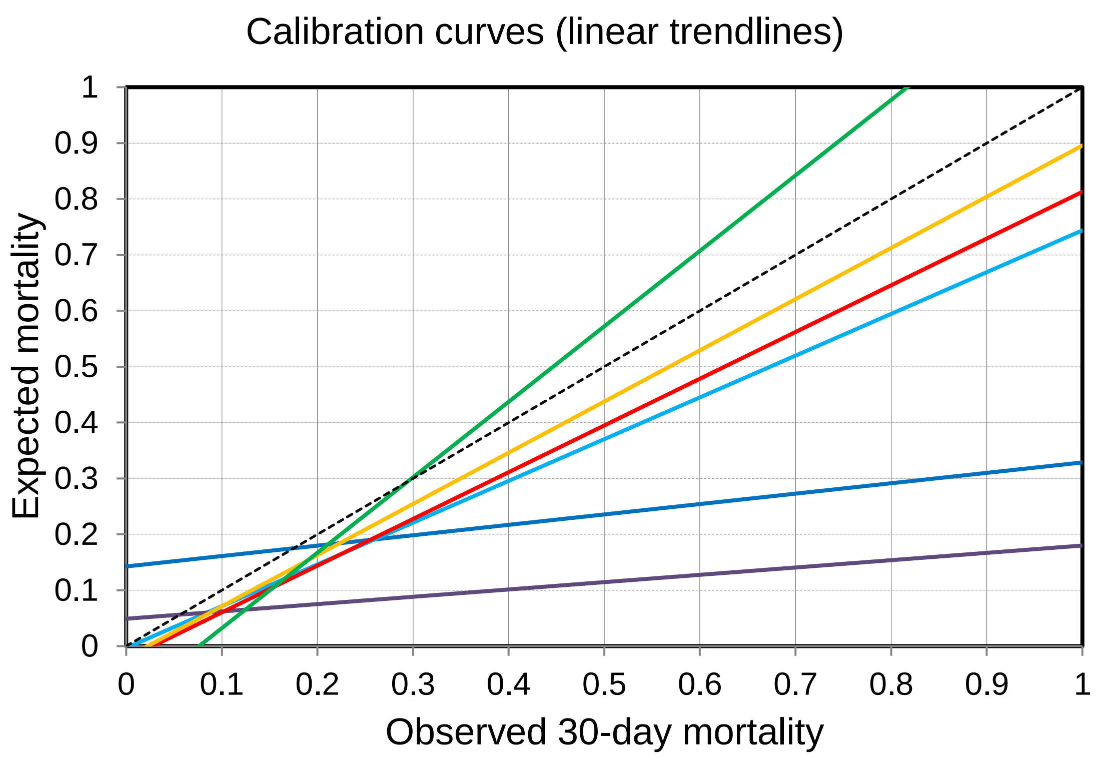

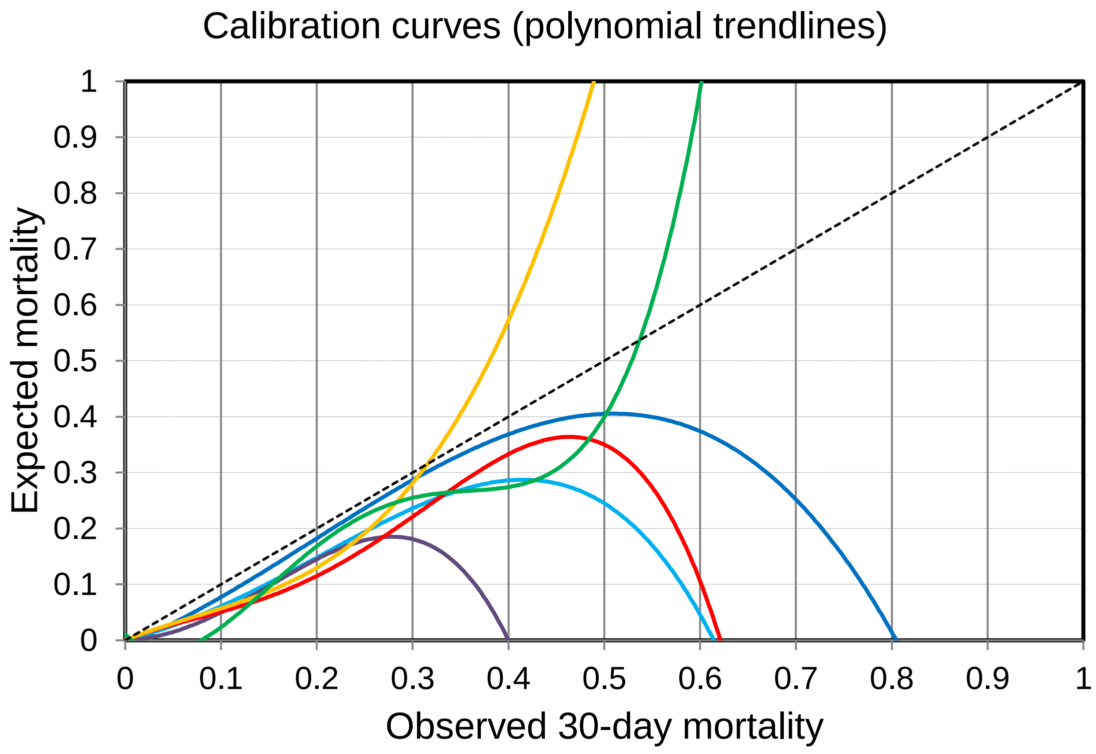

Suppl. Figure S2

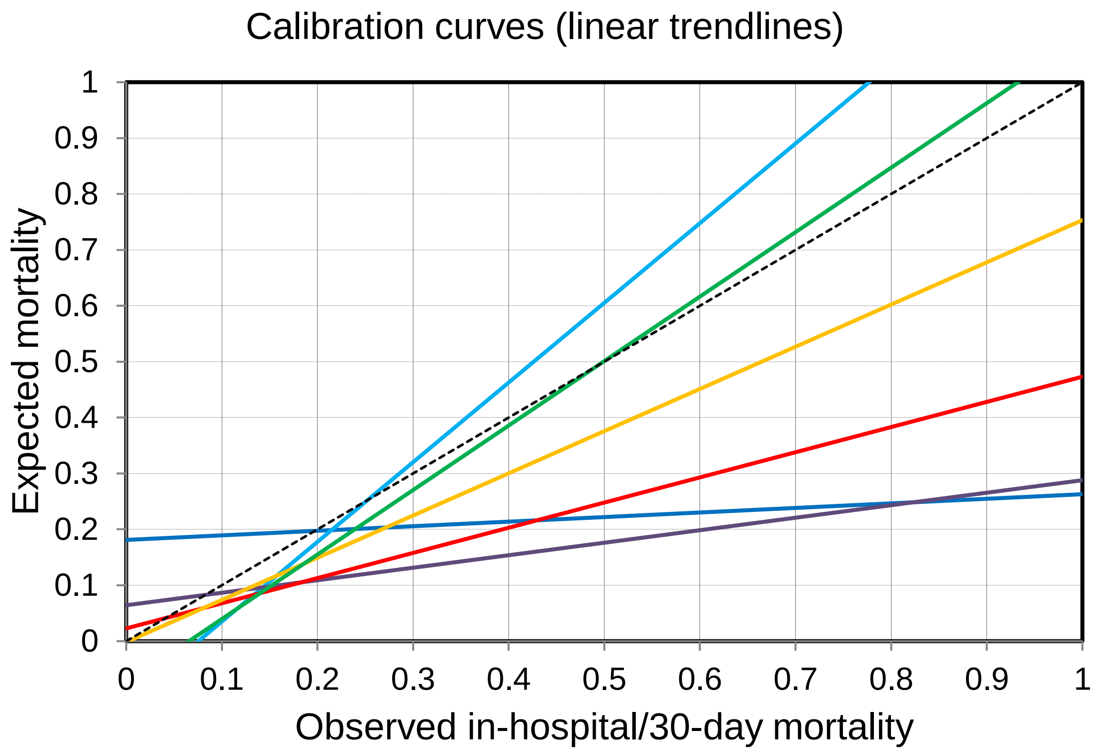

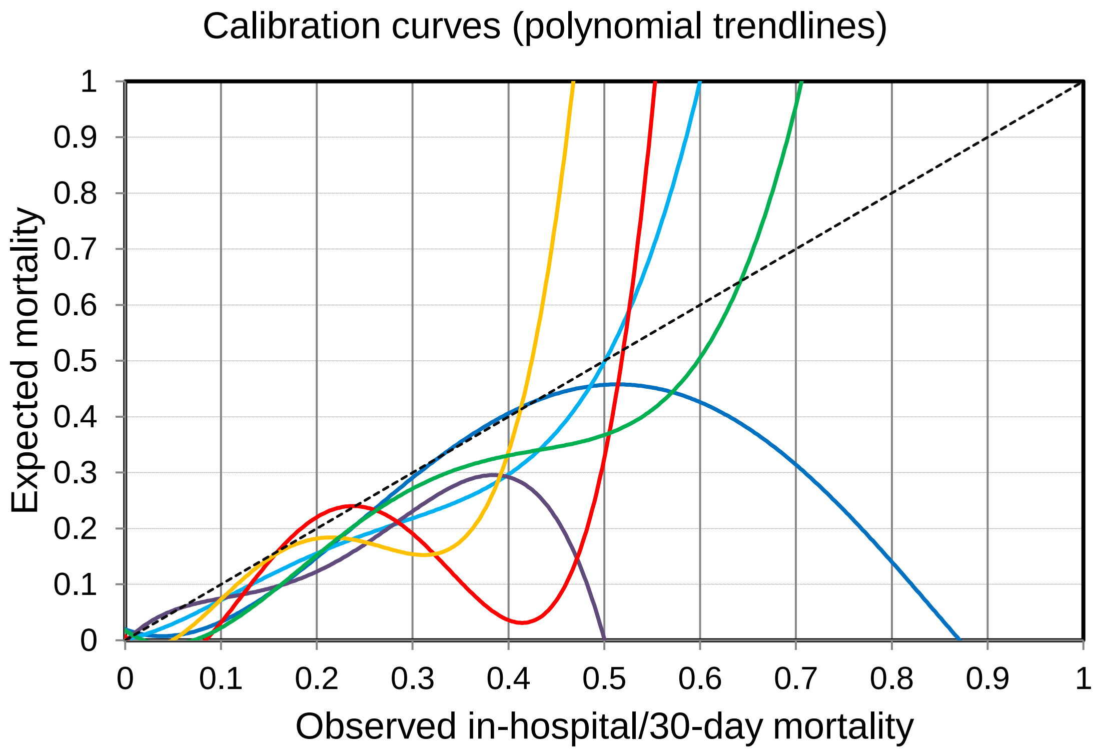

Suppl. Figure S2

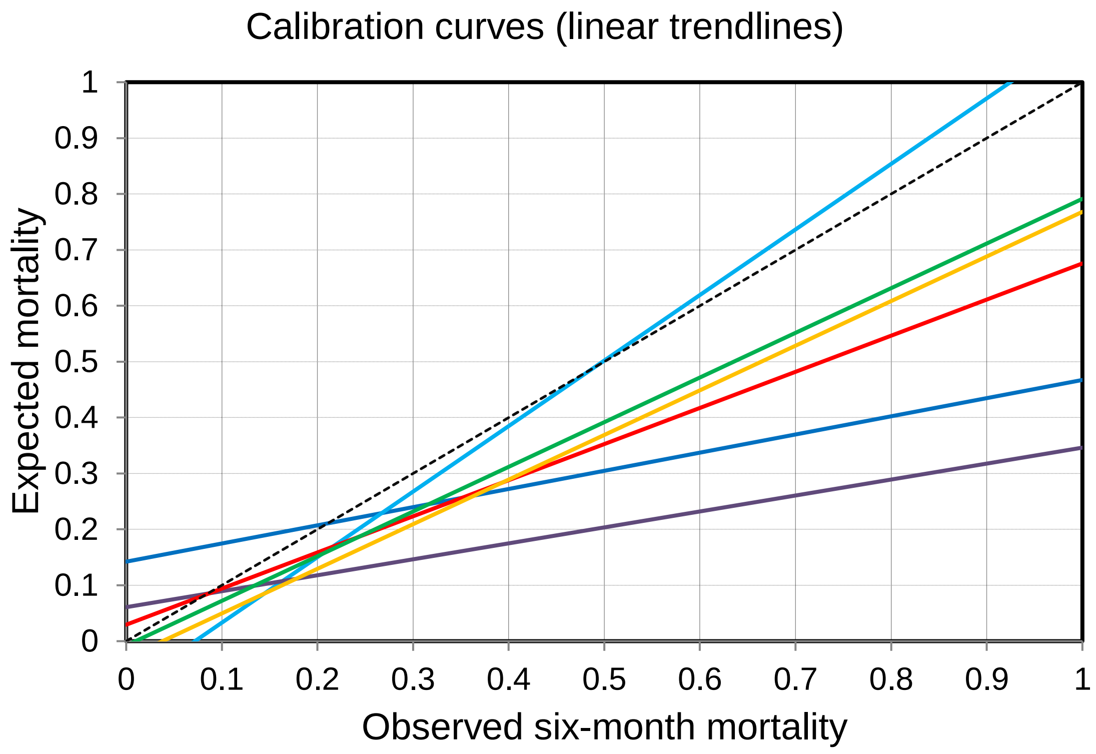

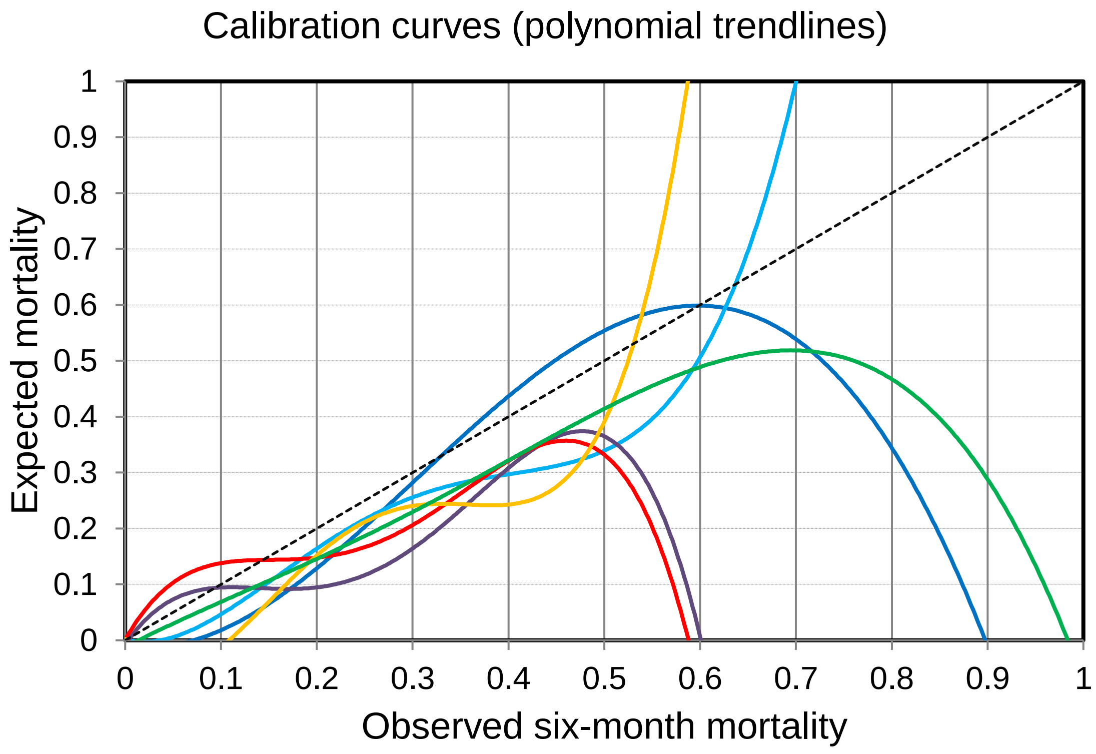

Suppl. Figure S2

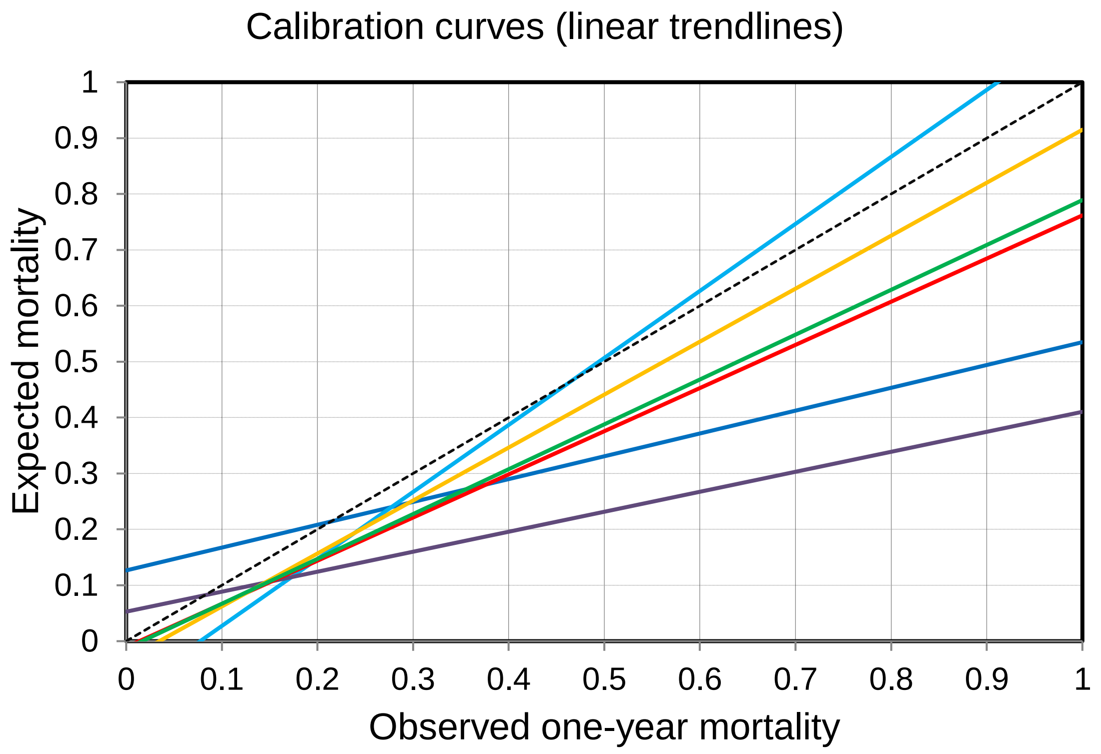

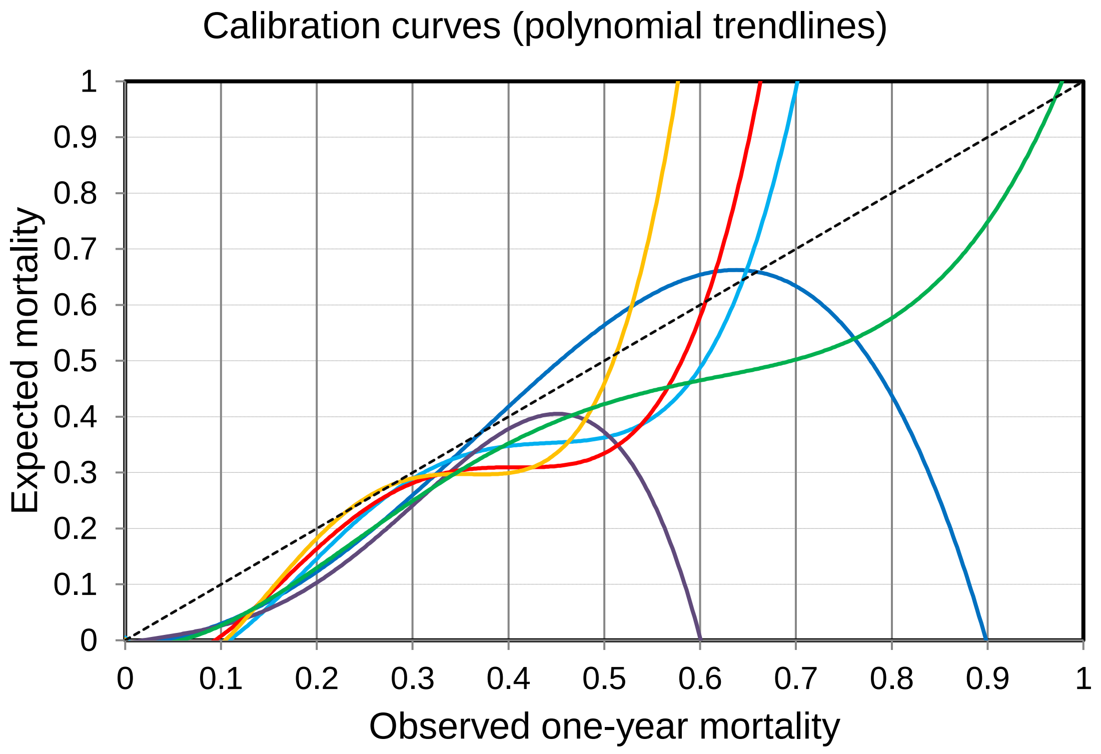

Supplement: Supplementary Figure S2 — Calibration curves for in-hospital, 30-day, in-hospital/30-day, six-month and one-year mortality after surgery for IE of six risk scores (N = 1,014). [file Image2.pdf]
