# Supplementary material for: Changing performance of surgical risk scores according to the endpoint of postoperative mortality in infective endocarditis patients
Source: Front Cardiovasc Med. 2025 Mar 13;12:1543049. doi: 10.3389/fcvm.2025.1543049 (PMC11965892; doi:10.3389/fcvm.2025.1543049)
Supplement: Supplementary Figure S3 — Receiver-operating characteristics (ROC) curves for in-hospital, 30-day, in-hospital/30-day, six-month and one-year mortality after surgery for IE of six risk scores (N = 1,014). [file Image3.pdf]

Supplementary Figure S3

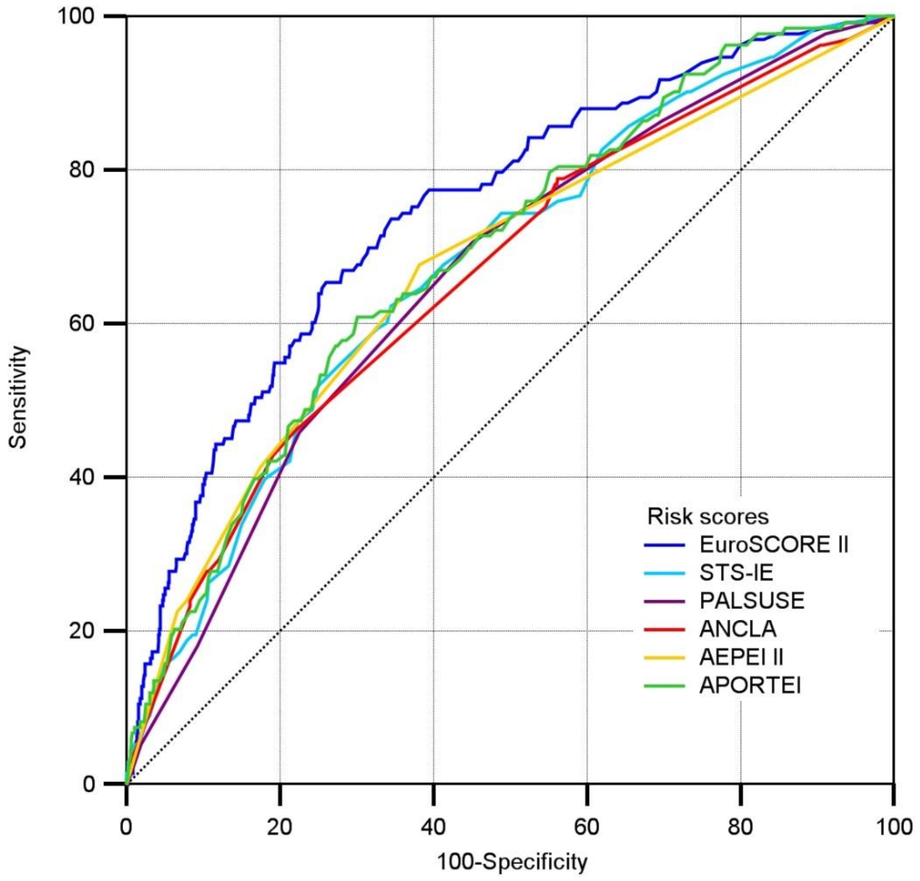

In-hospital mortality

Supplementary Figure S3

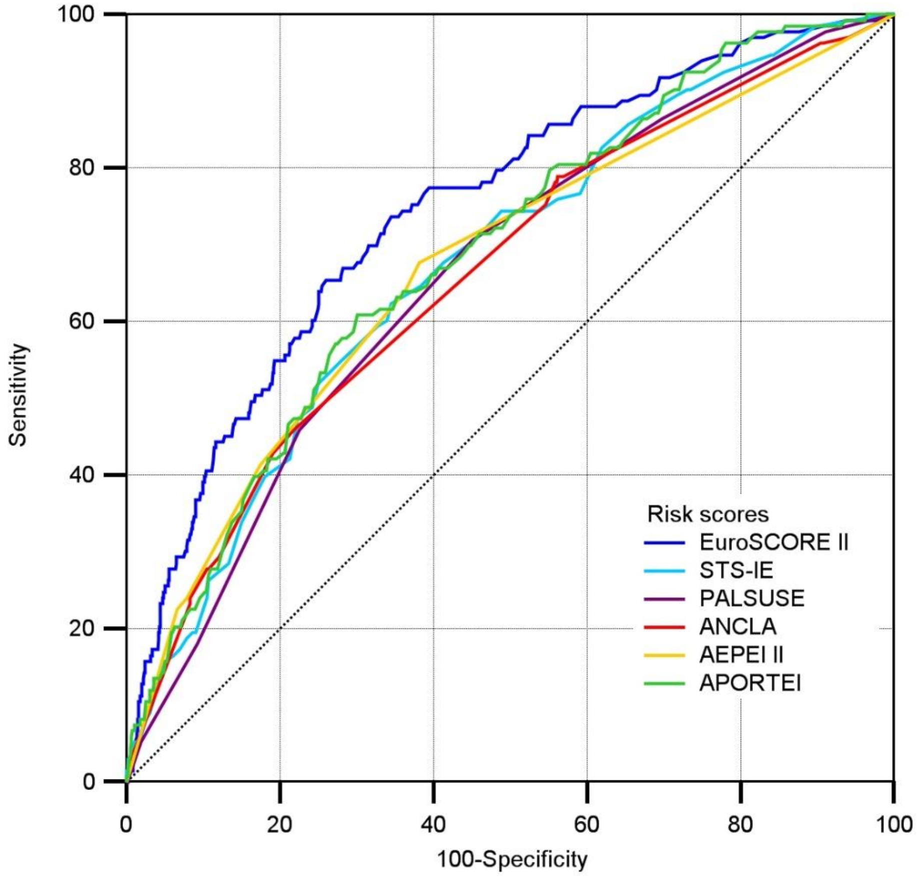

30-Day mortality

Supplementary Figure S3

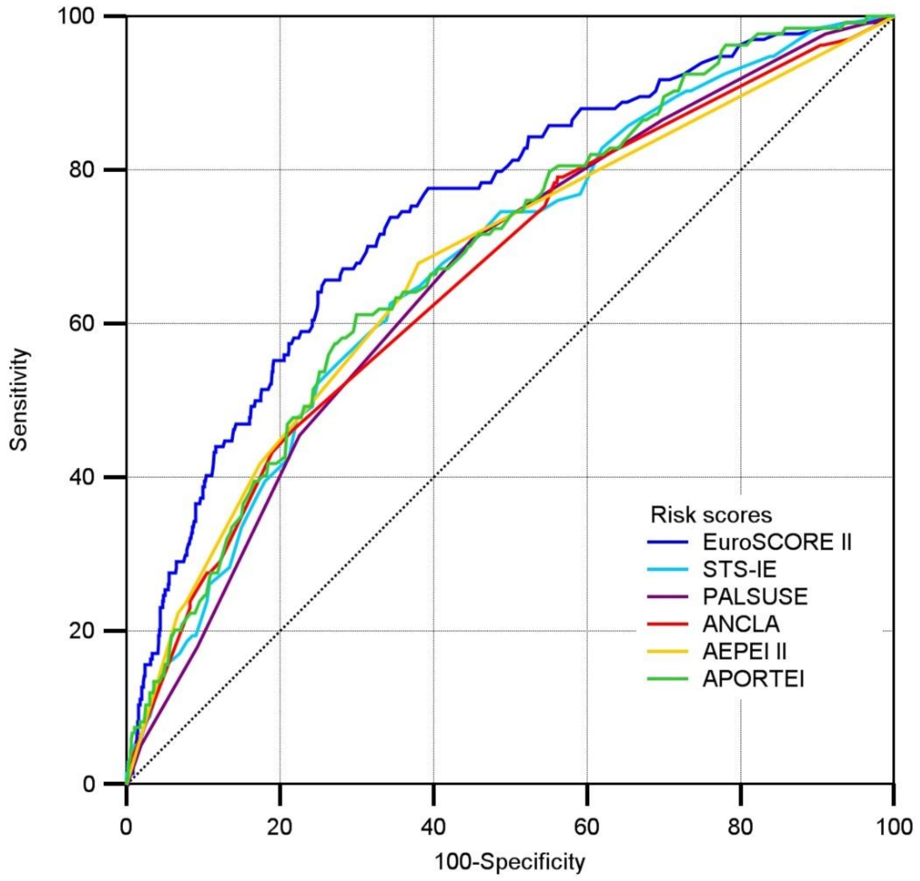

In-hospital/30-Day mortality

Supplementary Figure S3

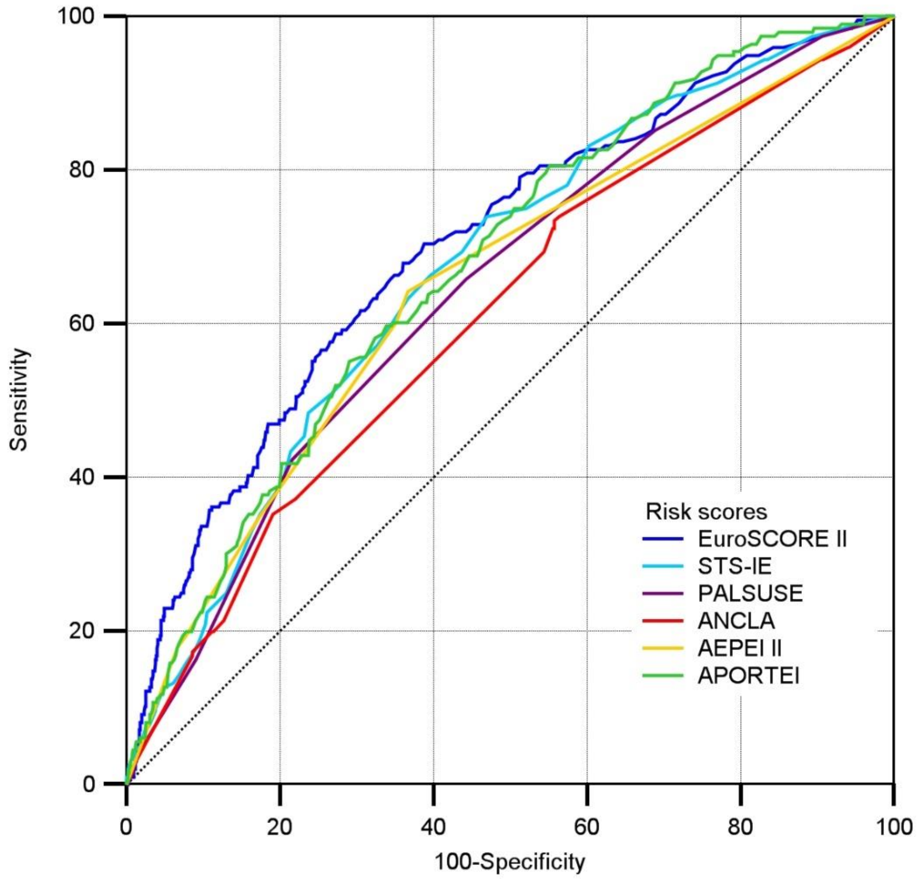

Six-month mortality

Supplementary Figure S3

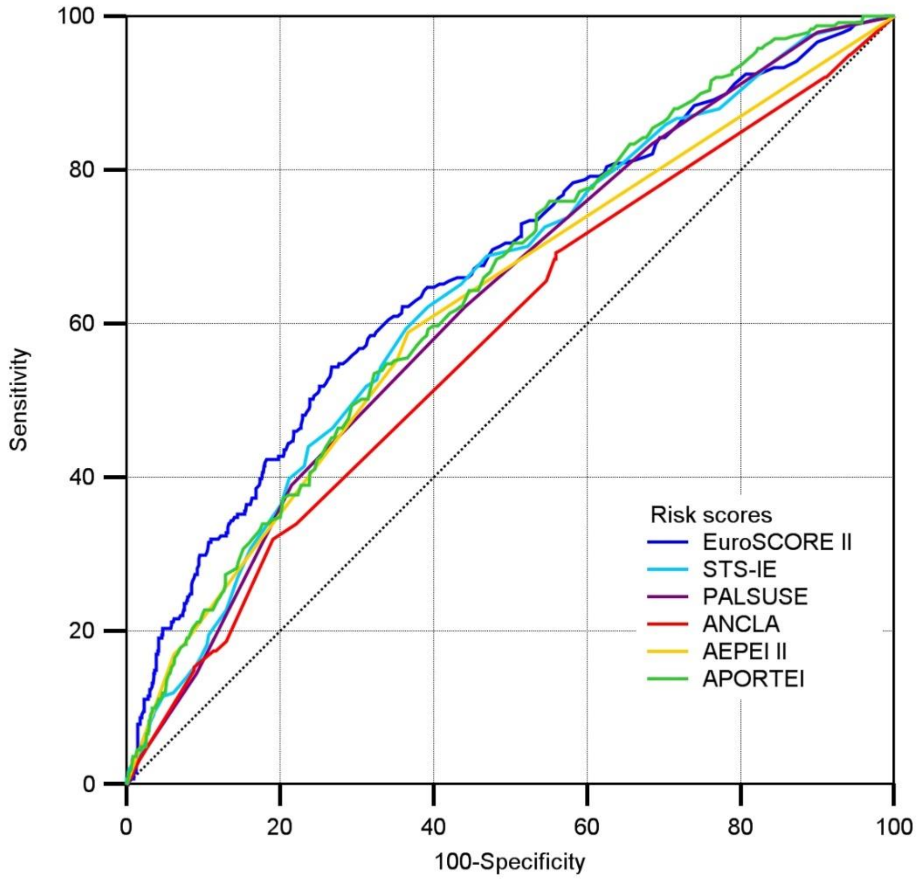

One-year mortality
