# Supplementary material for: Changing performance of surgical risk scores according to the endpoint of postoperative mortality in infective endocarditis patients
Source: Front Cardiovasc Med. 2025 Mar 13;12:1543049. doi: 10.3389/fcvm.2025.1543049 (PMC11965892; doi:10.3389/fcvm.2025.1543049)
Supplement: Supplementary file 4 [file Datasheet1.pdf]

Supplementary Tables

Supplementary Table S1: Specific risk scores for early mortality after surgery for IE: The characteristics of the corresponding derivation original studies*

Characteristic	Risk score				
	STS-IE†	PALSUSE‡	ANCLA§	AEPEI||	APORTEI**
Publication date	2011	2014	2017	2017	2019
First author	Gaca	Martinez-Sellés	Gatti	Gatti	Varela Barca
Type of study	Retrospective; Non-comparative	Retrospective; Comparative	Retrospective; Comparative	Retrospective; Comparative	Meta-analysis
Source	STS ACSD (824 centers in North America)	GAMES registry (26 Spanish hospitals)	University Hospital of Trieste, Italy	AEPEI registry (7 French centers) & Trieste University hospital, Italy	Sixteen studies on independent predictors of in-hospital death after surgery for IE
Period of patient enrollment	2002–2008 (7 yr)	2008–2010 (3 yr)	1999–2015 (17 yr)	2008 & 1999–2015 (1 yr + 17 yr)	2007–2018 (12 yr)
Derivation sample, No. of patients	13,617	437	138	361 (223+138)	7,484
Cardiac surgery patients, %	100	100	100	100	100
End-point	30-Day mortality	In-hospital mortality	In-hospital/30-day mortality	In-hospital/30-day mortality	In-hospital/30-day mortality
Statistical method	Multivariable analysis	Multivariable analysis	Multivariable analysis	Multivariable analysis	Systematic review (by

	(logistic regression)	(stepwise logistic regression)	(stepwise, backward logistic regression)	(stepwise, backward logistic regression)	PRISMA recommendations) on three databases; Eleven meta-analyses
Score design	Additive	Additive	Additive	Additive	Additive and logistic
Model	Preoperative-only	Preoperative-only	Preoperative-only; Combined	AEPEI I (original); AEPEI II (alternate)	Preoperative-only
Calibration	-	Hosmer-Lemeshow test	Hosmer-Lemeshow test; Contingency table	Hosmer-Lemeshow test; Contingency table	Hosmer-Lemeshow test; Calibration plot
Discrimination	AUC, 0.758	AUC, 0.73	AUC, 0.828 (preoperative-only) AUC, 0.823 (combined)	AUC, 0.780 (AEPEI I) AUC, 0.774 (AEPEI II)	AUC, 0.75
Classes of risk (score and expected mortality)	-	0, 0% >3, 45.4%	-	AEPEI II: 0–1, 19.6–34.1% 1.1–1.8, 36.6–47.7% 2.1–2.9, 55–68.3% 3.9, 82%	0–20, <10% 21–45, 10–20% 46–75, 20–40% >75, >40%
Internal validation	-	-	Bootstrapping (1,000 iterations)	Bootstrapping (1,000 iterations)	-
External validation sample,	5,926 (STS ACSD,	-	324 (Regione Autonoma	161 (AEPEI registry,	4,486 (GAMES registry,

No. of patients	2002–2008)		Friuli-Venezia Giulia, Italy, 1999–2018)	2001–2015)	34 Spanish hospitals, 2008–2018)
Comparison	-	Costa<br>De Feo-Cotrufo<br>Logistic EuroSCORE§§	STS-IE<br>DeFeo-Cotrufo<br>PALSUSE<br>OPR|||| Logistic EuroSCORE§§<br>EuroSCORE II	STS-IE†<br>DeFeo-Cotrufo<br>PALSUSE‡<br>OPR|||| Logistic EuroSCORE<br>EuroSCORE II	Logistic EuroSCOR
Other evaluations	-	-	-	Aikake information criterion;<br>EuroSCORE II vs. EuroSCORE II + BMI >27 kg/m2 (net reclassification index and integrated discrimination improvement)	QuIPS;<br>I^2 statistics;<br>Fixed- and random-effects model;<br>Forest plot graphs;<br>Egger method;<br>Funnel plot graphs;<br>Influence analysis;<br>Rob summary table

*IE defined according to the modified Duke criteria. *Ref.* 1.

†*Ref.* 2.

‡*Ref.* 3.

§*Ref.* 4.

||*Ref. 5*.

****Ref.** 6.

ACSD=Adult Cardiac Surgery Database; AEPEI=Association pour l'Étude et la Prévention de l'Endocardite Infectieuse; ANCLA=Anemia, NYHA class IV, Critical preoperative state, Large intra-cardiac destruction, surgery on thoracic Aorta; APORTEI=A*n*álisis de los factores PROnósticos en el Tratamiento quirúrgico de la Endocarditis Infecciosa; AUC=area under the Receiver-Operating Characteristic curve; EuroSCORE=European System for Cardiac Operative Risk Evaluation; GAMES=Grupo de Apoyo al Maneyo de la Endocarditis infecciosa en ESpana; IE=infective endocarditis; NYHA=New York Heart Association; PALSUSE=Prosthetic valve, Age ≥70, Large intra-cardiac destruction, Staphylococcus spp, Urgent surgery, Sex (female), EuroSCORE ≥10; PRISMA=Preferred Reporting Items for Systematic Reviews and Meta-Analyses; QuIPS=Quality in Prognostic Studies tool; STS=Society of Thoracic Surgeons

Supplementary Table S2: Specific risk scores for early mortality after surgery for definite IE: The corresponding variables*,†,‡

Class of variables	Risk score and variables				
	STS-IE§	PALSUSE‖	ANCLA<br>(preoperative-only<br>model)**	AEPEI II††	APORTEI‡‡
Host-related factors	Hypertension<br>[1.41; **5**]	Age, yr<br>[1.03]<br>≥70<br>[**1**]	NYHA class IV<br>[2.61; **1**]	NYHA class IV<br>[2.12; **1**]	Age<br>[1.03; **0.5x(age-50)**]
	Insulin-dependent<br>diabetes<br>[1.73; **8**]	Female sex<br>[2.1; **1**]	Critical preoperative<br>state<br>[4.97; **1.7**]	Critical preoperative<br>state<br>[2.35; **1.1**]	Female sex<br>[1.56; **7**]
	Non-insulin-dependent<br>diabetes<br>[1.54; **6**]	Urgent surgery<br>[2; **1**]			Previous cardiac surgery<br>[2.19; **13**]
	Chronic lung disease<br>[1.41; **5**]				NYHA class ≥III<br>[1.84; **10**]
	Prior CABG<br>[1.63; **7**]				Cardiogenic shock<br>[4.15; **24**]
	Prior valve surgery				Urgent surgery

	[1.63; **7**]				[2.39; **15**]
	Preoperative IABP or inotropes [1.96; **10**]				
	Urgent or emergency status, no cardiogenic shock [1.53; **6**]				
	Emergency, salvage, or cardiogenic shock [3.17; **17**]				
Heart-related factors	Multiple valve procedure [1.83; **9**]	Substantial intra-cardiac destruction [1.9; **1**]	Large intra-cardiac destruction [6.45; **1.9**]	-	Paravalvular abscess [2.39; **15**]
	Arrhythmia [1.66; **8**]	Prosthetic valve endocarditis [2.2; **1**]			Prosthetic endocarditis [1.98; **11**]
					Multivalvular involvement [1.35; **5**]

Extra-cardiac events	Renal failure or serum creatinine >2.0 mg/dL [2.29; **12**] Active endocarditis [2; **10**]	-	-	eGFR <50 mL/min [3.75; **1.8**]	Renal failure [2.57; **16**]
Pathogen-related factors	-	*Staphylococcus spp* [2.3; **1**]	-	-	*Staphylococcus aureus* [2.27; **14**]
Laboratory findings	-	-	Anemia [11; **2.5**]	-	-
Surgical data	-	-	Surgery on thoracic aorta [7.51; **2.1**]	-	-
Other	-	EuroSCORE ≥10% [**1**]	-	-	-

*IE defined according to the modified Duke criteria. *Ref.* 1.

†For each variable, the corresponding original odds ratio and/or the deriving assigned points (in bold) are reported in brackets (if disclosed).

‡For each risk score, the definitions of the adopted variables are reported in the corresponding original article.

§*Ref.* 2.

||*Ref.* 3.

***Ref.* 4.

††*Ref.* 5.

‡‡*Ref.* 6.

AEPEI=Association pour l’Étude et la Prévention de l’Endocardite Infectieuse; ANCLA=Anemia, NYHA class IV, Critical preoperative state, Large intra-cardiac destruction, surgery on thoracic Aorta; APORTEI=A*n*álisis de los factores PROnósticos en el Tratamiento quirúrgico de la Endocarditis Infecciosa; CABG=coronary artery bypass grafting; eGFR=estimated glomerular filtration rate; EuroSCORE=European System for Cardiac Operative Risk Evaluation; IABP=intra-aortic balloon pump; IE=infective endocarditis; NYHA=New York Heart Association; PALSUSE=Prosthetic valve, Age ≥70, Large intra-cardiac destruction, *Staphylococcus spp,* Urgent surgery, Sex (female), EuroSCORE ≥10; STS-IE=Society of Thoracic Surgeons-IE

Supplementary Table S3: Specific risk scores for early mortality after surgery for IE: The characteristics of the original datasets*

Variable	Risk score				
	STS-IE†	PALSUSE‡	ANCLA (preoperative-only model)§	AEPEI II‖	APORTEI**
No. of cases	13,617	437	138	361	7,484
Mortality					-
In-hospital	7.6%	24.3%	20.3%	15.5%	
30-Day	8.2%	-	17.4%	11%	
Age (mean ± SD) (yr)	55.1	61.4 ± 15.5	60.6 ± 8.5	59.1 ± 15.4	-.
>70		-	31.9%	26.3%	
Female gender	33.3%	24.7%	19.6%	21.1%	-.
BMI (mean ± SD) (kg/m^2)	-	-	25.5 ± 4.1;	25.4 ± 4.9	-
>30			13%	-	
BSA >2 m^2	37.6%	-	-	-	-
Arterial hypertension	56.2%	-	15.2%	30,7%	-
Diabetes mellitus	23.6%	24.3%	15.9%	19.4%	-
Insulin-dependent	11.1%	-	6.5%	7.5%	
Non-insulin-dependent	12.5%	-	9.4%	11.9%	
Chronic lung/obstructive-pulmonary disease	22.8%	16.9%	9.4%	9.1%	-
Chronic renal failure	23.3%	-	36.2%	33.5%	-

History of dialysis	13.3%		9.4%	4.2%	
Previous cardiac surgery	32.9%	-	26.8%	18.8%	-
CABG	7.9%				
Valve surgery	20.5%				
NYHA class					-
>I	49.8%	52.3%	81.9%	70.9%	
III-IV	-	-	-	-	
IV	-	-	39.9%	37.7%	
Cardiogenic shock/Use of inotropes or IABP	7.3%	-	19.6%	17.2%	-
Surgical priority		-			-
Urgent	49.8%		65.2%	51%	
Emergency/Salvage	7.3%		15.9%	12.1%	
Side of IE	-			-	-.
Isolated right		11%	8.7%		
Left or mixed		89%	91.3%		
Type of IE					-.
Native valve	79.5%	72.1%	80.4%	85%	
Prosthetic valve	20.5%	27.9%	19.6%	15%	
Valve involvement					-.
Aortic	35.7%	52.6%	62.3%	62%	

Mitral	40.2%	42.1%	43.5%	46.3%	
Tricuspid	4.1%	-	5.1%	9.7%	
Large intra-cardiac destruction					-.
Extensive valve damage	-	36.4%	23.2%	25.2%	
Perivalvular involvement	-	-	21.7%	29.6%	
Multiple valve involvement	19.9%	-	16.7%	21.6%	
Life-threatening arrhythmias	19.4%	-	-	-	-
LVEF (mean ± SD) (%)	-	-	56.6 ± 3.5%	56.6 ± 3.5;	-
<50			23.2%	19.9%	
<30			2.9%	1.4%	
PAP systolic (mean ± SD) (mmHg)	-	-	-	-	-
≥35			-	24.4%	
≥55			2%	5%	
Extra-cardiac events				-	-
Neurological dysfunction (recent)	5.6%	6.2%	9.4%		
Status of IE					-
Active	51.5%	100%	52.2%	76.2%	
Treated	48.5%	0	47.8%	23.8%	
Uncontrolled sepsis/Septic shock	-	7.6%	5.8%	-	-

Pathogen	-				-
Staphylococci spp		42.6%	23.9%	29%	
Staphylococcus aureus		-	16.7%	19.9%	
Streptococci spp		22.2	32.6%	37.1%	
Gram-negative spp		-	4.3%	4.2%	
Enterococci		-	9.4%	10.2%	
Fungi		-	1.4%	1.1%	
Other microorganisms		10%	-	0,1%	
Not identified		-	28.3%	18.3%	
Persistent bacteremia	-	18.1%	-	12.7%	-
Blood hemoglobin (g/dL)	-	-			-.
<12 for woman and 13 for man			81.9%	81.7%	
Blood platelets count ($\cdot 10^3/\mu L$)	-	-	221 ± 107	-	-.
<231			59.4%		
<150			-		
Serum peak C-reactive protein level (mean ± SD) (ng/dL)	-	-	203.8 ± 91.8	140.4 ± 85.5	-.
Surgery					-
Treatment	100%	100%	100%	100%	
On thoracic aorta	0.1%	-	6.5%	6.6%	
Aortic cross-clamp time (mean ± SD) (min)	-	-	122.9 ± 50.9	-	

>150	23.9%

*IE defined according to the modified Duke criteria. *Ref.* 1.

†*Ref.* 2.

‡*Ref.* 3.

§*Ref.* 4.

||*Ref.* 5.

***Ref.* 6.

AEPEI=Association pour l'Étude et la Prévention de l'Endocardite Infectieuse; ANCLA=Anemia, NYHA class IV, Critical preoperative state, Large intra-cardiac destruction, surgery on thoracic Aorta; APORTEI=A*n*álisis de los factores PROnósticos en el Tratamiento quirúrgico de la Endocarditis Infecciosa; CABG=coronary artery bypass grafting; eGFR=estimated glomerular filtration rate; EuroSCORE=European System for Cardiac Operative Risk Evaluation; IABP=intra-aortic balloon pump; IE=infective endocarditis; NYHA=New York Heart Association; PALSUSE=Prosthetic valve, Age ≥70, Large intra-cardiac destruction, *Staphylococcus spp,* Urgent surgery, Sex (female), EuroSCORE ≥10; STS-IE=Society of Thoracic Surgeons-IE
